# The Effectiveness of an App-Based Nurse-Moderated Program for New Mothers With Depression and Parenting Problems (eMums Plus): Pragmatic Randomized Controlled Trial

**DOI:** 10.2196/13689

**Published:** 2019-06-04

**Authors:** Alyssa Sawyer, Amy Kaim, Huynh-Nhu Le, Denise McDonald, Murthy Mittinty, John Lynch, Michael Sawyer

**Affiliations:** 1 School of Public Health University of Adelaide Adelaide Australia; 2 School of Medicine University of Adelaide Adelaide Australia; 3 Research and Evaluation Unit Women's and Children's Health Network Adelaide Australia; 4 Department of Psychology George Washington University Washington DC, DC United States; 5 Child and Family Health Service Women's and Children's Health Network Adelaide Australia; 6 Population Health Sciences University of Bristol Bristol United Kingdom

**Keywords:** mobile phone, infant, mother-child relations, postnatal depression, randomized controlled trial

## Abstract

**Background:**

Postnatal depression and caregiving difficulties adversely affect mothers, infants, and later childhood development. In many countries, resources to help mothers and infants are limited. Online group–based nurse-led interventions have the potential to help address this problem by providing large numbers of mothers with access to professional and peer support during the postnatal period.

**Objective:**

This study tested the effectiveness of a 4-month online group–based nurse-led intervention delivered when infants were aged 2 to 6 months as compared with standard care outcomes.

**Methods:**

The study was a block randomized control trial. Mothers were recruited at the time they were contacted for the postnatal health check offered to all mothers in South Australia. Those who agreed to participate were randomly assigned to the intervention or standard care. The overall response rate was 63.3% (133/210). Primary outcomes were the level of maternal depressive symptoms assessed with the Edinburgh Postnatal Depression Scale (EPDS) and quality of maternal caregiving assessed using the Parenting Stress Index (PSI; competence and attachment subscales), the Parenting Sense of Competence Scale (PSCS), and the Nursing Child Assessment Satellite Training Scale. Assessments were completed at baseline (*mean child age* 4.9 weeks [*SD* 1.4]) and again when infants were aged 8 and 12 months.

**Results:**

Outcomes were evaluated using linear generalized estimating equations adjusting for postrandomization group differences in demographic characteristics and the outcome score at baseline. There were no significant differences in the intervention and standard care groups in scores on the PSI competence subscale (*P*=.69) nor in the PSCS (*P*=.11). Although the group by time interaction suggested there were differences over time between the EPDS and PSI attachment subscale scores in the intervention and standard care groups (*P*=.001 and *P*=.04, respectively), these arose largely because the intervention group had stable scores over time whereas the standard care group showed some improvements between baseline and 12 months. Mothers engaged well with the intervention with at least 60% (43/72) of mothers logging-in once per week during the first 11 weeks of the intervention. The majority of mothers also rated the intervention as helpful and user-friendly.

**Conclusions:**

Mothers reported that the intervention was helpful, and the app was described as easy to use. As such, it appears that support for mothers during the postnatal period, provided using mobile phone technology, has the potential to be an important addition to existing services. Possible explanations for the lack of differences in outcomes for the 2 groups in this study are the failure of many mothers to use key components of the intervention and residual differences between the intervention and standard care groups post randomization.

**Trial Registration:**

Australian New Zealand Clinical Trials Registry ACTRN12616001732471; http://www.ANZCTR.org.au/ACTRN12616001732471.aspx (archived on WebCite as http://www.webcitation.org/77zo30GDw)

## Introduction

Postnatal depression causes significant distress for mothers and is associated with a range of adverse outcomes for children. There is good evidence that caregiving difficulties associated with depressive symptoms play a key role in mediating the association between maternal depression and child outcomes [1-6]. For example, postnatal depression has the potential to negatively affect breastfeeding, infant sleep routines, and attendance at health checks, all of which can adversely affect children’s later growth and development [4].

More recently it has been recognized that subthreshold postnatal depressive symptoms can also affect maternal functioning and infant development [6-8]. This has led many countries, including Australia, to initiate universal screening programs for early identification of mothers with depressive symptoms. However, a major challenge for screening programs is the paucity of clinical services available to support mothers with comorbid depressive symptoms and parenting problems and the difficulty of engaging busy and/or isolated new mothers with treatment programs [9].

Greater use of the internet to deliver services to mothers during the postnatal period offers a potential solution to these problems. Internet use by women of childbearing age in Australia and other countries is now ubiquitous with new mothers making extensive use of the internet to obtain child-raising information and social support [10-12]. This has encouraged the development of a vast array of websites and *mobile phone apps* by commercial, professional, and government organizations. However, as noted by Plantin and Daneback [10], health-related information on the internet can be misleading and occasionally, *utterly wrong* [10,13]. In addition, there is a marked absence of evaluations assessing whether online information and support can improve maternal and child outcomes.

The intervention evaluated in this study was developed as a response to the unresolved issue about how services can effectively address the high prevalence of mild-to-moderate depression and associated parenting problems experienced by many women during the postnatal period [14-16]. The study also responds to the National Institute for Health and Care Excellence guidelines calling for randomized controlled trials (RCTs) designed to evaluate the effectiveness of interventions to help mothers with depressive symptoms and parenting difficulties [1,6,7]. The latter is important as the subthreshold levels of postnatal depressive symptoms in the early postnatal period are a risk factor for the development of postnatal depression and also have the potential to interfere with optimal mother-infant development [6,17,18]. Importantly, an RCT is pragmatic and delivered within a state-wide child and family health service system. This approach was adopted to enhance the likelihood that translation into new service models would occur if the intervention was effective, as compared with efficacy trials conducted by researchers external to real-world service delivery systems.

The intervention extends earlier research by our group which evaluated an online group–based nurse-led intervention designed to help mothers in the general population better manage common parenting problems experienced by mothers during the postnatal period [19-21]. The enhanced intervention reported here used a 4-month online group–based nurse-led approach to provide help with both postnatal depressive symptoms and parenting problems for mothers who were identified as having comorbid mild-to-moderate levels of depressive symptoms and problems caring for their infants during the first 4 weeks after the birth of their infant.

The evaluation compared maternal and infant outcomes for those who received the new internet-based intervention versus outcomes for those who received standard postnatal home- and clinic-based support from a community nurse, routinely offered to all mothers in South Australia (Australian New Zealand Clinical Trials Registry ACTRN12616001732471). The primary outcomes were (1) the level of maternal depressive symptoms, (2) the quality of maternal caregiving including the level of parenting self-competence, and (3) the quality of the mother-infant relationship, assessed when infants were aged 8 and 12 months [22].

## Methods

### Participants, Recruitment, and Randomization

Participants were new mothers referred by their birthing hospital for their initial postnatal health check by nurses based at one of 13 Child and Family Health Service (CaFHS) community clinics located in Adelaide or 1 clinic located in a large regional center in South Australia. From March to June 2017, when nurses visited mothers for maternal postnatal health checks (undertaken when infants are aged 1 to 4 weeks), they asked mothers if they would give permission for the research team to contact them by telephone if the mother was eligible to participate in the study. Eligibility criteria were (1) Edinburgh Postnatal Depression Scale (EPDS) score ≥7; (2) at least 1 self-reported parenting problem; (3) literacy in English; and (4) access to a smartphone.

**Figure 1:**
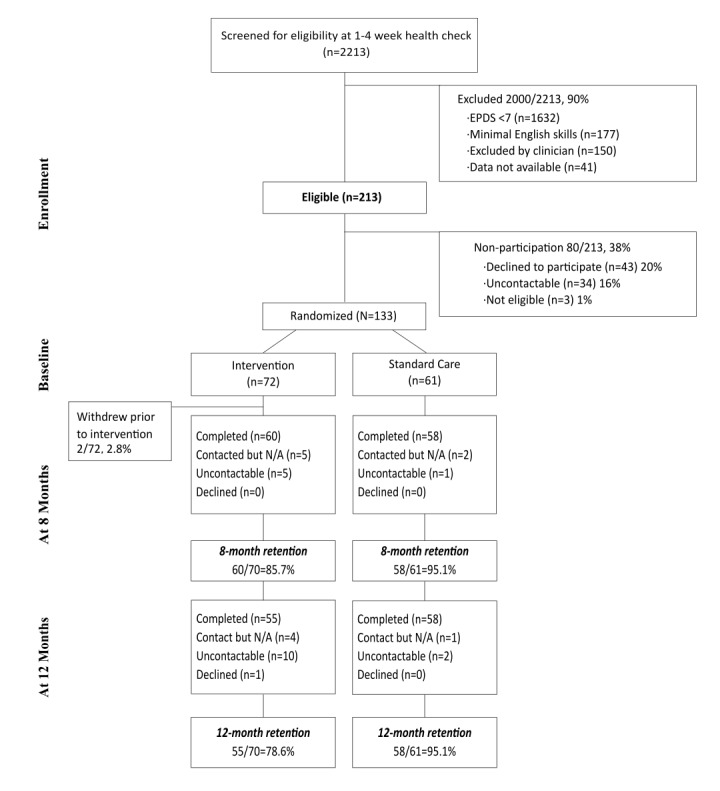
Flow chart of participants.

In total, 2213 mothers agreed to be contacted by telephone and subsequently completed a nurse-provided questionnaire comprised of a 4-item parenting problem scale and the EPDS [23]. Among these mothers, 1632 mothers had EPDS scores <7, 177 had minimal English skills, and 150 mothers were not included as they were identified by nurses as having high levels of distress and, consistent with clinical practice guidelines, were referred for additional support [24]. There were a further 41 mothers who were identified by nurses as not being eligible for the study, but as a result of an administrative error, the reason for noneligibility was not provided to the research team. This left 213 mothers who scored >7 on the EPDS *and* reported at least 1 problem on the 4-item parenting problem questionnaire. A member of the research team telephoned these mothers to obtain verbal consent for a home visit by a study field worker. Among these mothers, 34 could not subsequently be contacted by telephone, 43 declined to participate in the study when contacted by telephone, and 3 were found to be ineligible. This left 133 mothers who completed the baseline assessment and were randomly assigned to the intervention or standard care arms of the study (Figure 1). Written consent was obtained at the time of this preintervention home visit.

Full details of the research design are provided in the trial protocol [22]. In brief, the trial utilized a block randomized control design. Blocks of 20 consecutive mothers were randomized to either the intervention arm or the comparison (*standard care*) arm of the study using a randomization schedule that was generated by a statistician who was independent of the study team. The research team was blind to group allocation at the time of recruitment and assignment of mothers to the study groups. However, owing to the nature of the intervention, after the intervention commenced, it was not possible to keep the research staff or field workers blind to the groups to which mothers had been allocated. The exception to this was research staff coding the Nursing Child Assessment Satellite Training (NCAST) Parent-Child Interaction scale, who were blind to group allocation while completing coding. Further information regarding the randomization process is provided in the paper describing the trial protocol [22].

### Intervention Versus Standard Care

**Figure 2:**
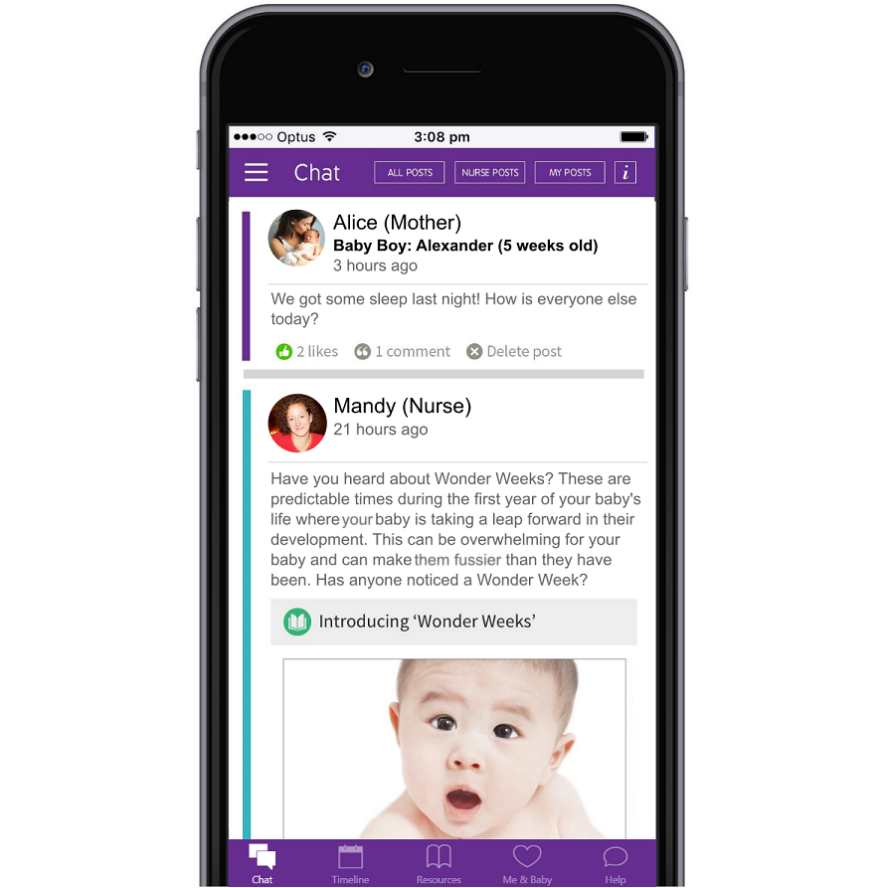
The eMums Plus mobile phone app.

Mothers randomized to the intervention arm of the study were assigned to a nurse-led, online group consisting of approximately 20 mothers of similarly aged infants. The 4-month intervention was delivered when infants were aged approximately 2 to 6 months and was accessed by mothers via a mobile phone app (see Figure 2). The app was free to download from the relevant mobile app store (ie, iTunes or Google Play), and mothers accessed the app on their own personal mobile devices (mobile phones or tablets). Nursing staff delivering the intervention were trained in the use and management of the app. They also received additional training in the mental health components of the intervention. [22]

Full details of the intervention are provided in the manuscript describing the trial protocol [22]. New mothers and CaFHS nurses were involved in the development of the intervention. In addition, new mothers participating in CaFHS face-to-face mothers’ groups were utilized to test the usability of the intervention app, with iterative improvements made before its use in the study, based on their advice.

Mothers in the comparison arm received *standard care*. In South Australia, standard care consists of a single home visit by a CaFHS nurse, usually within 4 weeks of the infant’s birth. The nurse checks the health of the mother and baby, provides general advice about infant care, and provides information about community services available to help mothers and infants. Mothers in the intervention arm could also access community services if they wished to do this. Mothers in the intervention arm were not paid for their participation; however, a “thank you” gift was posted to the child of the participating mother upon completion of each questionnaire (3 in total).

### Measures

Trained field workers administered self-complete questionnaires to mothers in their homes. Field workers also video-recorded mothers completing the NCAST teaching interaction during home visits. All measures, including the NCAST, were completed when infants were aged 1 to 2 months (preintervention), 8 months, and 12 months.

### Primary Outcomes

#### Maternal Depressive Symptoms

The level of depressive symptoms experienced by mothers was assessed using the EPDS. The EPDS is a 10-item self-report questionnaire that assesses symptomatology during the previous 7 days. Scores range from 0 to 30 with higher scores indicating higher levels of depressive symptoms. Cut-off points can also be used to identify mothers who may benefit from additional support [23].

#### Maternal Self-Competence

1. Mothers’ perceptions of their parental efficacy and their satisfaction with the parenting role were assessed using the 16-item Parenting Sense of Competence Scale (PSCS). Scores range from 16 to 96 with higher scores indicating higher levels of parenting self-competence and satisfaction with their parenting role [25,26].
2. The Parenting Stress Index (PSI) Competence subscale (excluding 2 items assessing parental education) was used to assess mothers’ perception of their competence in caring for their infant [27]. The scale consisted of 11 items with scores ranging from 11 to 55. Higher scores indicated lower self-competence.

#### Quality of Mother-Infant Relationship

1. The NCAST Scale was used to assess the quality of mother-child interactions. The NCAST utilizes a 3 to 5-min video-recording of mothers teaching their child a skill appropriate to the age of their child. For the purpose of this study, we used the Teaching Scale as it is suitable for use with infants aged 0 to 36 months. Mother-child interactions are assessed in 6 areas: sensitivity to cues, response to distress, fostering social-emotional functioning, fostering cognitive growth, clarity of cues, and responsiveness to the caregiver [28]. Trained research assistants coded the video-recordings to generate a total score for mothers (including the subscales’ sensitivity to cues, response to distress, fostering social-emotional functioning, and fostering cognitive growth) and a total score for infants (including the subscales’ clarity of cues and responsiveness to the caregiver) [28]. Higher scores indicate higher levels of positive mother-child interaction quality. Further details about the NCAST scores are included in the Statistical Analyses section below.
2. The PSI Attachment subscale was used to assess mothers’ perceptions of the quality of their relationship with their infant [27]. The scale consisted of 7 items with scores ranging from 7 to 35. Higher scores indicated the lower quality of mother-infant bonding.

The approach of using more than one measure to rate primary outcomes was employed to determine the consistency of the results, regardless of the particular measure used to assess the outcome. The analysis of results was undertaken independently for each measure.

### Secondary Outcomes

1. Service utilization: Maternal self-completed questionnaires were used to identify other online or face-to-face health services used by mothers and infants.
2. Intervention quality: At the 8-month assessment, mothers completed a 40-item questionnaire designed by the researchers to assess mothers’ perceptions of the quality of the intervention. Items in the questionnaire asked about the helpfulness of the intervention and the usability of the mobile phone app.
3. App usage: Data about app usage were automatically collected and included the number of log-ins, comments, and replies that mothers posted and the number of times mothers accessed the different elements of the intervention available on the app.

### Ethics Approval

Ethics approval was received from the Women’s and Children’s Health Network Human Research Ethics Committee (approval numbers SSA/16/WCHN/016, HREC/16/WCHN/014).

### Missing Data

In total, 22 participants did not complete the 8-month assessment (*N*=2), the 12-month assessment (*N*=7), or both assessments (*N*=13). We compared demographic characteristics and levels of functioning at baseline of these 22 participants and those with complete data at all assessments (*N*=111). Compared with mothers who completed all assessments, those who missed an assessment were (1) younger (missed assessment mean 28.4 years vs completed assessment mean 31.7 years; *95* % *CI* −5.4 to −1.2); (2) had delivered their first child (missed assessment percentage=90.9% (20/22) vs completed assessment percentage=61.3% (68/111) *;* *P*=.01); (3) less likely to have university qualifications (missed assessment percentage=40.9% (9/22) vs completed assessment percentage=64.9% (72/111) *;* *P*=.02); and (4) had lower EPDS scores at their baseline assessment (missed assessment mean 7.2 vs completed assessment mean 9.1; *95* % *CI* −3.7 to −0.1).

Multiple imputations could not be performed owing to low cell sizes in the main predictors of missingness. We also attempted to calculate inverse probability response weights to account for the missing data, but weights could not be calculated owing to low cell sizes. As a result, we have presented complete case analyses in the main paper and analyses for all mothers with at least 1 outcome score at 8 or 12 months in the supplementary tables (see Multimedia Appendices 1 and 2) [29].

A small number of items were not completed on some measures. Where this occurred, missing item scores were estimated on the basis of mean item scores for the measure, as recommended by instructions for the measure.

### Statistical Analyses

All analyses were intention-to-treat. Initially, unadjusted results were examined. Subsequently, adjusted mean scores for intervention and standard care groups at each time point were compared using linear generalized estimating equations) using exchangeable within-group correlation structures [30]. In each model, predictor variables were group (intervention and standard care), time (baseline, 8 months, and 12 months), and a group-by-time interaction. Analyses were adjusted for residual differences in demographic characteristics after randomization including the number of children, maternal education, housing situation, maternal age (years) at baseline, and the outcome score at baseline. All analyses were conducted using STATA 15.1 (StataCorp) [31].

There were 4 online mothers’ groups in the study. However, intraclass correlations were very small at both 8- and 12-month assessments, ranging from 0.00 to 0.01 for the study outcomes. This indicated that there was little clustering of outcomes within groups. As a result, we did not adjust for clustering in the analysis of results.

A number of problems were identified with the NCAST videos recorded during the baseline and 8-month assessments. These included, for example, a failure to record the start of the teaching interactions; interruptions by field workers during teaching interactions (such as providing additional instructions or speaking to the child); and a failure to record the mother’s or baby’s face during some of the interaction tasks. These problems have the potential to invalidate NCAST scoring. After the problems were identified, further training was provided for field workers. As a result, the problems were largely overcome by the time of the 12-month assessment. However, given the problems with earlier assessments, we have only included results from the 12-month NCAST assessment in this study.

At the 12-month assessment, a small number of items were coded as *missing* for a large percentage of mothers because it was difficult to record the required mother-child behavior. As a result, items that could not be coded for more than 12% (13/113) of participants were not included in the NCAST score. In total, scores from 1 item were not included in the mother total score (“the caregiver provides non-verbal feedback to the child after the child performs better than the last attempt”) and 4 items were not included in the child total score (“the child smiles/laughs during the episode”, “the child grimaces/frowns during the episode”, “the child smiles at caregiver within five seconds after caregiver’s verbalization”, and “the child smiles at caregiver within five seconds after caregiver’s gesture, touch or facial expression”). As such, the mother total score consisted of 49 items (rather than 50) with possible scores ranging between 0 and 49. The child total score consisted of 19 items (rather than 23) with possible scores ranging between 0 and 19.

A linear regression analysis was used to compare the intervention versus standard care group NCAST scores for mothers and infants at the 12-month assessment. The scores were adjusted for the number of children, maternal education, housing situation, and maternal age (years) at baseline.

The frequency with which mothers logged on to the intervention, their frequency of message posting, and the extent to which they utilized different elements of the intervention are described using means and median scores. The number of additional services (both online and face-to-face services) that mothers used to support them in caring for themselves or their infants was assessed using percentage scores.

### Sample Size

The sample size target for this study was 160 (80 in each trial arm). This sample size would provide .80 power to detect an effect size of Cohen *d*=0.4 at alpha=.05.

## Results

The demographic characteristics and baseline scores for mothers in the complete case samples are shown in Table 1. The demographic characteristics and baseline scores for mothers in the response samples are shown in Multimedia Appendix 2.

Compared with mothers in the standard care group, mothers in the intervention group more frequently had more than 1 child, were younger, lived in rental accommodation, and had less frequently completed tertiary education. They also reported lower scores on the PSI competence subscale and higher scores on the PSCS and lower scores on the PSI attachment subscale (indicating better functioning on all 3 measures). A larger number of mothers in the intervention group than the standard care group could not be contacted at the time of follow-up assessments (Figure 1). It is possible that this was due to mothers in the intervention group being a more mobile population as reflected in their higher frequency of living in rental accommodation. All these postrandomization differences between the groups were adjusted for in the analysis of results.

The unadjusted outcome scores are presented in Table 2. Mothers in the intervention group had lower PSI competence scores and higher PSCS scores (indicating better functioning on both measures). They also had lower PSI attachment scores (also indicating better functioning). There was little difference in the EPDS scores between the intervention and standard care groups, except at the 12-month assessment where, on average, mothers in the standard care group had lower EPDS scores than mothers in the intervention group.

**Table 1:** Baseline demographic characteristics of children and mothers in the complete case samples in the intervention and standard care groups.

Characteristics		Complete case				*P* value^a^
		Intervention		Standard care		
		Values	n	Values	N	
First child, n (%)		29 (54)	54	39 (68)	57	.11
Male child, n (%)		29 (54)	54	27 (47)	57	.51
Child indigenous, n (%)		2 (4)	54	0 (0)	57	.14
Single parent household, n (%)		1 (2)	54	2 (4)	57	.59
Maternal age (years), mean (SD)		31.1 (5)	54	32.2 (4)	57	.16
**Mother’s education^b^, n (%)**						
	University degree	28 (52)	54	44 (77)	57	.008
	Trade or technical school	20 (37)	54	7 (12)	57	.008
	Some or all years of high school	6 (11)	54	6 (11)	57	.008
**Mother’s employment, n (%)**						
	Full-time paid employment	32 (59)	54	36 (63)	57	.51
	Part-time paid employment	16 (30)	54	18 (32)	57	.51
	Other (self-employed or casual)	3 (6)	54	0 (0)	57	.51
	Unemployed	3 (6)	54	3 (5)	57	.51
**Housing, n (%)**						
	Rental or other	24 (44)	54	15 (26)	57	.046
	Own home	30 (56)	54	42 (74)	57	.046
**Currently breastfeeding, n (%)**						
	Yes	46 (85)	54	51 (90)	57	.5
	No	8 (15)	54	6 (11)	57	.5
**Partner’s education^b,c^, n (%)**						
	University degree	21 (40)	53	26 (47)	55	.63
	Trade or technical school	20 (38)	53	20 (36)	55	.63
	Some or all years of high school	12 (23)	53	9 (16)	55	.63
**Partner’s employment^c^, n (%)**						
	Full-time paid employment	46 (87)	53	46 (84)	55	.49
	Part-time paid employment	2 (4)	53	5 (9)	55	.49
	Other (self-employed, contract, or casual)	3 (6)	53	4 (7)	55	.49
	Unemployed	2 (4)	53	0 (0)	55	.49
**Parenting stress index^d^, mean (SD)**						
	Competence	26.5 (5.2)	54	28.8 (5.8)	57	.03
	Attachment	10.9 (2.9)	54	13.6 (4.8)	57	.001
**Maternal caregiving, mean (SD)**						
	Parenting Sense of Competence Scale	67.4 (10.3)	54	61.6 (9.7)	57	.003
Edinburgh Postnatal Depression Scale, mean (SD)		8.8 (3.0)	54	9.5 (4.9)	57	.38

^a^For the comparison between the intervention and standard care groups for the complete case sample.

^b^Highest level of completed education.

^c^Note that the single parents in the intervention and standard care conditions did not report their partner’s education or employment; as a result, the complete case sample has fewer n values in these cells, but these parents are not excluded.

^d^For the Parenting Stress Index, higher scores indicate a worse outcome.

**Table 2:** Unadjusted mean outcome scores and mean difference (95% CI) between scores in the intervention and standard care groups.

Outcome assessment			Intervention (N=54)	Standard care (N=57)	Mean difference (95% CI)
**Maternal confidence**					
	**Parenting Stress Index (PSI) competence^a^**				
		Baseline	26.5	28.8	2.3 (0.2 to 4.4)
		8 months	24.4	25.8	1.5 (−0.7 to 3.7)
		12 months	23.6	25.2	1.6 (−0.5 to 3.8)
**Parent Sense of Competence Scale**					
	Baseline		67.6	61.6	−5.7 (−9.5 to −1.9)
	8 months		69.2	64.9	−4.4 (−7.9 to −0.9)
	12 months		69.5	67.3	−2.2 (−5.9 to 1.6)
**Relationship quality**					
	**PSI attachment^a^**				
		Baseline	10.9	13.6	2.7 (1.1 to 4.2)
		8 months	10.4	12.5	2.2 (0.9 to 3.5)
		12 months	10.9	12.1	1.2 (−0.1 to 2.6)
**Maternal Depression**					
	**Edinburgh Postnatal Depression Scale**				
		Baseline	8.8	9.5	0.7 (−0.9 to 2.2)
		8 months	7.9	8.7	0.7 (−1.1 to 2.5)
		12 months	8.6	7.0	−1.5 (−3.2 to 0.1)

^a^Higher scores indicate more problems.

The adjusted outcome scores are shown in Table 3. There was little difference in the adjusted mean PSI competence scores between the groups at any of the assessments. However, at the baseline assessment, the adjusted mean Parent Sense of Competence score was higher for mothers in the intervention group (indicating better functioning) than for mothers in the standard care group. In contrast, there was little difference in the adjusted mean Parent Sense of Competence scores across the groups at the time of the 8-month and 12-month assessments.

The adjusted mean PSI attachment score was lower for mothers in the intervention group (indicating better quality relationships) than mothers in the standard care group at both the baseline and 8-month assessments but differed by a little across the groups at the 12-month assessment. There were only small differences in the adjusted mean EPDS scores between the groups at each assessment.

In the standard care group but not the intervention group, Parenting Sense of Competence scores were higher at the 12-month assessment than at baseline and EPDS scores were lower (indicating better functioning on each questionnaire).

A possible explanation for the improved functioning in the standard care group was that mothers in the group accessed more additional health services than mothers in the intervention group. We investigated this possibility by reviewing responses to questions asking about the frequency with which mothers in each group accessed face-to-face or online health services. There were few significant differences in service use between the groups but mothers in the intervention group had more frequently visited their family doctor and also a hospital emergency department *2 or more times* during the previous 6 months at the 12-month assessment (family doctor: intervention group=78% (42/54), standard care group=63% (36/57), *P*=.09; hospital emergency department: intervention group=15% (8/54), standard care group=4% (2/54), *P*=.04). They had also more frequently accessed online resources such as the *Baby Centre* website at the 8-month assessment (weekly/daily—intervention group=41% (22/54), standard care group=21% (12/56), *P*=.03) and the *Red nose* website or app at the 12-month assessment (monthly/weekly/daily—intervention group=67% (36/54), standard care group=46% (26/57), *P*=.03).

There was no difference in either the mother or infant adjusted mean NCAST total scores between the intervention and standard care groups (Table 4).

**Table 3:** Adjusted mean (95% CI) outcome scores in the intervention (N=54) and standard care (N=57) groups. All scores are adjusted for the number of children, maternal education, housing situation, and maternal age (years) at baseline.

Outcome assessment			Intervention, mean (95% CI)	Standard care, mean (95% CI)	Group × time, *P* value^a^
**Maternal confidence**					
	**Parenting Stress Index (PSI) competence^b^**				
		Baseline	26.5 (25.2 to 27.9)	28.8 (27.2 to 30.4)	.69
		8 months	24.4 (22.7 to 26.1)	25.8 (24.4 to 27.2)	.69
		12 months	23.6 (22.2 to 24.9)	25.2 (23.6 to 26.8)	.69
**Parent Sense of Competence Scale**					
	Baseline		67.2 (64.5 to 69.9)	61.8 (59.1 to 64.4)	.11
	8 months		69.1 (66.5 to 71.7)	64.9 (62.8 to 67.2)	.11
	12 months		69.4 (66.8 to 72.0)	67.4 (64.7 to 70.1)	.11
**Relationship quality**					
	**PSI attachment^b^**				
		Baseline	10.9 (10.1 to 11.8)	13.6 (12.4 to 14.9)	.04
		8 months	10.3 (9.4 to 11.2)	12.6 (11.6 to 13.5)	.04
		12 months	10.9 (9.9 to 11.7)	12.1 (11.1 to 13.1)	.04
**Maternal Depression**					
	**Edinburgh Postnatal Depression Scale**				
		Baseline	8.6 (7.7 to 9.5)	9.6 (8.3 to 10.9)	.001
		8 months	7.8 (6.6 to 9.0)	8.8 (7.5 to 10.1)	.001
		12 months	8.4 (7.2 to 9.6)	7.2 (5.9 to 8.3)	.001

^a^Statistical significance of the group × time interaction.

^b^Higher scores indicate more problems.

**Table 4:** Adjusted Nursing Child Assessment Satellite Training mean (95% CI) scores for intervention and standard care groups in the complete case sample. All scores are adjusted for the number of children, maternal education, housing situation, and maternal age (years) at baseline.

NCAST^a^ assessment at 12 months		Intervention		Standard care		Statistics, B^b^ (95% CI)
		Mean (95% CI)	N	Mean (95% CI)	N	
**Mother total score**						
	Unadjusted	36.5 (35.6 to 37.3)	51	36.2 (35.3 to 37.2)	54	—^c^
	Adjusted	36.6 (35.7 to 37.5)	51	36.1 (35.2 to 37.0)	54	0.47 (−0.85 to 1.80)
**Child total score**						
	Unadjusted	15.5 (15.1 to 15.9)	51	15.5 (14.9 to 15.9)	52	—
	Adjusted	15.5 (15.0 to 15.9)	51	15.4 (14.9 to 15.9)	52	0.06 (−0.66 to 0.78)

^a^NCAST: Nursing Child Assessment Satellite Training; note that owing to administration errors, the n value varies slightly from the full complete case sample.

^b^Unstandardized regression coefficient.

^c^Not applicable.

### Percentage of Mothers Logging Into the Intervention, Posting Messages, and Using Each Element of the Intervention

In the first 11 weeks of the intervention, more than 60% (43/72) of participants logged into the intervention at least once each week (the first table in Multimedia Appendix 3). Furthermore, more than 50% (38/72) of mothers logged into the intervention at least once each week until the 14th week of the 16-week intervention. This suggests that most mothers were regularly engaging with the intervention for the vast majority of the 16 weeks in which it was delivered. A somewhat lower percentage of mothers were regularly posting messages on the Chat page (the first table in Multimedia Appendix 3). However, almost 50% (36/72) of mothers posted at least 1 message each week until the 6th week of the 16-week intervention. Subsequent to this, the percentage of mothers posting at least 1 message each week varied, but even in the 10th week, 50% (36/72) of mothers posted at least 1 message.

The intervention app had a number of components that provided mothers with different kinds of information and support (see Multimedia Appendix 4). Full details about these elements are available in the paper describing the study protocol [22]. The second table in Multimedia Appendix 3 shows the percentage of mothers who accessed each component at least once per week. The Chat page where mothers could communicate with each other and where nurses posted comments and responded to queries was the component most frequently accessed (the second table in Multimedia Appendix 3). In contrast, after the initial weeks of the intervention, mothers less frequently accessed the other app components, including the resources component that contained key intervention content designed to support parenting and maternal emotional health (the second table in Multimedia Appendix 3).

### Qualitative Assessment of the Intervention by Mothers

At the 8-month assessment (after they had completed the intervention), mothers were asked if they had received sufficient information to effectively use the app features. In total, 80% (47/59) of mothers *strongly agreed* or *agreed* that they had received sufficient information to use the Chat and Resources components of the app whereas 68% (40/59) to 76% (45/59) *strongly agreed* or *agreed* that they had received sufficient information to use each of the other app components.

At the 8-month assessment, mothers were also asked to rate the helpfulness and user-friendliness of each component of the intervention. In all areas, the majority of mothers reported that intervention components were *very helpful* or *helpful* (the third table in Multimedia Appendix 3). With the exception of mood graphing and video components of the app, the majority of mothers who used each component also reported that it was *very easy* or *easy* to use (the fourth table in Multimedia Appendix 3). However, a large percentage (30% (18/60) to 47% (28/60)) of mothers did not use some key components of the app such as the *mood-rater* designed to allow mothers to track their mood level over time and activities embedded in topic areas that were designed to help improve maternal emotional health (the fourth table in Multimedia Appendix 3). Finally, 90% (52/58) of mothers reported that the length of the information in each topic area was *about right*. In addition, 44% (25/57) of mothers reported the length of the intervention was *about right*, whereas 51% (29/57) reported that it was *too short*.

## Discussion

### Principal Findings

This study examined the effectiveness of a 4-month online group–based intervention led by community child health nurses and delivered via a mobile phone app. The intervention aimed to improve outcomes for mothers experiencing depressive symptoms and difficulty caring for their infants.

When infants were aged 8 and 12 months, maternal ratings of their level of maternal depressive symptoms and their parenting competence did not differ across the intervention and standard care groups. Standard care provided to mothers in each group consisted of a single home visit by a CaFHS nurse, within 4 weeks of the infant’s birth, to check the health of the mother and baby and to provide information about relevant community services for mothers and infants. When infants were aged 12 months, there was also no difference between the observed quality of mother-infant interactions in the intervention versus standard care groups. The lack of differences between the groups occurred despite mothers in the intervention group engaging well with the intervention, logging into the app regularly, and posting comments more frequently than has been reported with previous online interventions [32,33]. In addition, a high percentage of mothers in the intervention group reported that the app was user-friendly, and its components were helpful for them.

There are 2 possible explanations for the findings from the study. First, although most mothers in the intervention group regularly logged into the app and communicated with each other through the Chat page, they made much less use of the app’s text-based resources designed to provide support for depressive symptoms and guidance about how to solve common parenting problems. Furthermore, mothers also made little use of the activities in each topic area that were designed to reinforce skills being addressed by the topic. As such, it is possible that although most mothers reported that the intervention was helpful and the app easy to use, a failure to fully utilize key components in the resource meant that the intervention did not produce measurable improvements in maternal depressive symptoms and maternal parenting competence, beyond those achieved with standard care.

Second, despite mothers being randomly assigned to the intervention and standard care groups, the intervention group contained a higher proportion of socially disadvantaged mothers than the standard care group. For example, at the baseline assessment a higher percentage of mothers in the intervention group were living in rental accommodation and had lower levels of educational achievement than mothers in the standard care group. It is possible that the greater level of social disadvantage among mothers in the intervention group made them less likely to benefit from a largely self-directed online group–based intervention. It is also possible that these differences contributed to a higher level of attrition among mothers in the intervention group owing to the research team being unable to relocate a larger number of mothers in the intervention group than in the standard care group.

One of the key motivations for examining the effectiveness of nurse support and an intervention provided online through a mobile phone app is the limited availability of traditional face-to-face services to support the large number of mothers who experience difficulties during the postnatal period. The aim was to use innovative app-based technology to increase the number of mothers with depressive symptoms and parenting problems who can be provided with support by existing health services. Internet delivery has the potential to allow nurses to provide ongoing support services without the need to travel to mothers’ homes, reduce costs of *no-show* visits, and allow one nurse to have contact with many mothers during a single day. For mothers, internet delivery has the potential to enable access to reliable, evidence-based *just-in-time* information along with both professional and peer support without the need to attend fixed-time appointments in clinics that may be geographically distant from their homes.

The results of the study suggest that during the postnatal period, mothers will engage and utilize support provided through a mobile phone app. They also suggest that mothers find this method of receiving support to be helpful and user-friendly. As such, it appears that support for mothers during the postnatal period provided using mobile phone technology has the potential to be an important addition to existing services. However, based on results in this study, a continuing challenge is to find ways of getting mothers to engage with key elements of online interventions to ensure that they receive a sufficient *dosage* of the intervention components required to reduce depressive symptoms and improve parenting skills.

### Strengths and Limitations

One of the main strengths of the study was its pragmatic nature. The RCT was conducted within a routine service setting using existing administrative infrastructure to allocate participants and existing clinical staff to deliver the intervention as part of normal service delivery. As a result, the intervention is able to be taken up as a part of routine delivery by the service and the results are those that we can expect if the service takes up this intervention. A potential limitation of this study is that residual differences in demographic characteristics between the intervention and standard care groups existed despite randomization [34]. The intervention group had characteristics associated with a higher level of disadvantage that may have led to experiences of greater adversity throughout the intervention that could have affected their levels of depression and mother-infant interaction quality independently of the intervention and in ways that differed to mothers in the standard care group.

### Conclusions

Large numbers of women experience comorbid depressive symptoms and difficulties with caregiving during the postnatal period. However, there are limited health services available to support these women and their infants. In particular, there is a lack of services that provide combined support for both depression and caregiving difficulties during this period of time.

The eMums intervention did not reduce depressive symptoms nor improve maternal caregiving. Changes to outcome scores on the EPDS and PSI attachment scale suggested there was some improvement in these areas for mothers in the standard care group, largely after the intervention had ended. However, these changes were very small and unlikely to be of clinical significance.

Mothers in the intervention group reported that the intervention was helpful, and the app was described as easy to use. As such, this method of treatment delivery has the potential to support larger numbers of mothers than is possible using clinic-based face-to-face treatments. The major challenge will be to find ways to ensure that participating mothers fully engage with all the active intervention components required to reduce their depressive symptoms and improve their parenting skills.

## Appendix

Multimedia Appendix 1 Results for participants with outcome scores complete for a least one assessment postintervention.

Multimedia Appendix 2 Baseline demographic characteristics of children and mothers for the response sample in the intervention and standard care groups.

Multimedia Appendix 3 Use of the intervention and mothers’ reports of the quality of different elements of the intervention.

Multimedia Appendix 4 Key components of the eMums intervention app.

Multimedia Appendix 5 CONSORT-EHEALTH checklist (V 1.6.1).

## Abbreviations

CaFHS: Child and Family Health Service
EPDS: Edinburgh Postnatal Depression Scale
NCAST: Nursing Child Assessment Satellite Training
NHMRC: National Health and Medical Research Council
PSCS: Parenting Sense of Competence Scale
PSI: Parenting Stress Index
RCT: randomized controlled trial
