# Supplementary material for: The Effectiveness of an App-Based Nurse-Moderated Program for New Mothers With Depression and Parenting Problems (eMums Plus): Pragmatic Randomized Controlled Trial
Source: J Med Internet Res. 2019 Jun 4;21(6):e13689. doi: 10.2196/13689 (PMC6682297; doi:10.2196/13689)
Supplement: Multimedia Appendix 1 [file jmir_v21i6e13689_app1.docx]

**Table 1.** Adjusted mean (SE), and difference between mean (95% CI) outcome scores for intervention (n=61) and standard care (n=59) groups with outcome data from at least one follow up assessment^a^. All scores adjusted for number of children, maternal education, housing situation, and maternal age (years) at baseline.

| Outcome Assessment | Intervention | Standard care | Group x time |
| --- | --- | --- | --- |
|  | M (SE), 95% CI | M (SE), 95% CI | *P value* |
|  |  |  |  |
| **Maternal Confidence** |  |  |  |
| PSI Competence^a^ |  |  | .60 |
| Baseline | 26.3 (0.6), 25.0 – 27.6 | 28.9 (0.8), 27.3 – 30.4 | - |
| 8 months | 24.3 (0.8), 22.8 – 25.8 | 26.0 (0.7), 24.6 – 27.4 | - |
| 12 months | 23.5 (0.6), 22.2 – 24.8 | 25.2 (0.8), 23.7 – 26.8 | - |
|  |  |  |  |
| **Parent Sense of Competence Scale** |  |  | .12 |
| Baseline | 66.8 (1.3), 64.3 – 69.2 | 62.1 (1.3), 59.5 – 64.6 | - |
| 8 months | 68.8 (1.2), 66.4 – 71.1 | 65.1 (1.1), 63.0 – 67.3 | - |
| 12 months | 69.0 (1.3), 66.4 – 71.5 | 67.6 (1.3), 65.0 – 70.2 | - |
|  |  |  |  |
| **Relationship Quality** |  |  |  |
| PSI Attachment^b^ |  |  | .06 |
| Baseline | 11.0 (0.4), 10.3 – 11.8 | 13.4 (0.6), 12.2 – 14.7 | - |
| 8 months | 10.5 (0.4), 9.7 – 11.4 | 12.5 (0.5), 11.6 – 13.4 | - |
| 12 months | 11.0 (0.4), 10.1 – 11.8 | 12.0 (0.5), 11.1 – 13.0 | - |
|  |  |  |  |
| **Maternal Depression** |  |  |  |
| EPDS |  |  | .001 |
| Baseline | 8.4 (0.4), 7.6 – 9.2 | 9.6 (0.7), 8.3 – 10.9 | - |
| 8 months | 7.9 (0.6), 6.8 – 9.0 | 8.8 (0.6), 7.6 – 10.1 | - |
| 12 months | 8.3 (0.6), 7.2 – 9.4 | 7.1 (0.6), 6.0 – 8.3 | - |

Abbreviations: CI = confidence interval, PSI, Parenting Stress Index; SE, standard error.

^a^ Participants had complete baseline demographic data and at least one outcome assessment (this is the minimum requirement for an individual to be included in the GEE analyses). All scores adjusted for baseline demographic characteristics as described in the manuscript.

^b^ Higher scores indicate more problems.
