# Supplementary material for: The Effectiveness of an App-Based Nurse-Moderated Program for New Mothers With Depression and Parenting Problems (eMums Plus): Pragmatic Randomized Controlled Trial
Source: J Med Internet Res. 2019 Jun 4;21(6):e13689. doi: 10.2196/13689 (PMC6682297; doi:10.2196/13689)
Supplement: Multimedia Appendix 2 [file jmir_v21i6e13689_app2.docx]

**Table 1.** Baseline demographic characteristics of children and mothers for the response sample in the intervention and standard care groups.

| Characteristic | Response sample | | | |
| --- | --- | --- | --- | --- |
|  | Intervention | | Standard care | |
|  | M (SD) or % | n | M (SD) or % | n |
|  |  |  |  |  |
| First child, n (%) | 45 (62.5) | 72 | 43 (70.5) | 61 |
| Male child, n (%) | 36 (50.0) | 72 | 29 (47.5) | 61 |
| Child Indigenous, n (%) | 2 (2.8) | 72 | 1 (1.6) | 61 |
| Single parent household, n (%) | 2 (2.8) | 72 | 3 (4.9) | 61 |
| Maternal age, mean (SD) | 30.5 (4.7) | 72 | 31.8 (4.6) | 61 |
|  |  |  |  |  |
| **Mother’s education^a^, n (%)** |  | 72 |  | 61 |
| University degree | 36 (50.0) |  | 45 (73.8) |  |
| Trade or technical school | 25 (34.7) |  | 8 (13.1) |  |
| Some or all years of high school | 11 (15.3) |  | 8 (13.1) |  |
|  |  |  |  |  |
| **Mother’s employment, n (%)** |  | 72 |  | 61 |
| Full-time paid employment | 43 (59.7) |  | 38 (62.3) |  |
| Part-time paid employment | 21 (29.2) |  | 19 (31.2) |  |
| Other (self-employed or casual) | 2 (2.8) |  | - |  |
| Unemployed | 6 (8.3) |  | 4 (6.6) |  |
|  |  |  |  |  |
| **Housing, n (%)** |  | 72 |  | 61 |
| Rental or other | 32 (44.4) |  | 16 (26.2) |  |
| Own home | 40 (55.6) |  | 45 (73.8) |  |
|  |  |  |  |  |
| **Currently breastfeeding, n (%)** |  | 72 |  | 61 |
| Yes | 61 (84.7) |  | 53 (86.9) |  |
| No | 11 (15.3) |  | 8 (13.1) |  |
|  |  |  |  |  |
| **Partner’s education^ab^, n (%)** |  | 70 |  | 58 |
| University degree | 25 (35.7) |  | 28 (48.3) |  |
| Trade or technical school | 26 (37.1) |  | 20 (34.5) |  |
| Some or all years of high school | 19 (27.1) |  | 10 (17.2) |  |
|  |  |  |  |  |
| **Partner’s employment^b^, n (%)** |  | 70 |  | 58 |
| Full-time paid employment | 61 (87.1) |  | 49 (84.5) |  |
| Part-time paid employment | 3 (4.3) |  | 5 (8.6) |  |
| Other (self-employed, contract, or casual) | 2 (2.9) |  | 3 (5.2) |  |
| Unemployed | 4 (5.7) |  | 1 (1.7) |  |
|  |  |  |  |  |
| **Parenting Stress Index^c^, M (SD), 95%CI** |  |  |  |  |
| Competence | 26.1 (5.1),  24.9 – 27.3 | 72 | 28.9 (5.7),  27.4 -30.3 | 61 |
| Attachment | 11.1 (3.2),  10.4 – 11.9 | 72 | 13.4 (4.8),  12.1 – 14.6 | 61 |
|  |  |  |  |  |
| **Maternal caregiving, M (SD), 95%CI** |  |  |  |  |
| Parenting Sense of Competence Scale | 66.9 (9.9),  64.7 – 69.3 | 72 | 61.9 (9.6),  59.4 – 64.3 | 61 |
|  |  |  |  |  |
| **Edinburgh Postnatal Depression Scale, M (SD), 95%CI** | 8.3 (3.2),  7.5 – 9.0 | 72 | 9.4 (4.8),  8.2 – 10.7 | 61 |

^a^ Highest level of completed education

^b^ Note that the single parents in the intervention and standard care conditions did not report partner’s education or employment as a result the complete case sample has fewer n in these cells but these parents are not excluded.

^c^ For the Parenting Stress Index higher scores indicate a worse outcome.
