# Supplementary material for: The Effectiveness of an App-Based Nurse-Moderated Program for New Mothers With Depression and Parenting Problems (eMums Plus): Pragmatic Randomized Controlled Trial
Source: J Med Internet Res. 2019 Jun 4;21(6):e13689. doi: 10.2196/13689 (PMC6682297; doi:10.2196/13689)
Supplement: Multimedia Appendix 3 [file jmir_v21i6e13689_app3.docx]

**Table 1**. Percentage of mothers who logged into the app at least once each week and the percentage of mothers who posted at least once each week (n=72)

| Intervention week | Percentage | |
| --- | --- | --- |
|  | Logins | Posts |
|  |  |  |
| 1 | 78 | 64 |
| 2 | 78 | 69 |
| 3 | 67 | 46 |
| 4 | 75 | 50 |
| 5 | 68 | 46 |
| 6 | 69 | 49 |
| 7 | 68 | 44 |
| 8 | 61 | 39 |
| 9 | 68 | 33 |
| 10 | 68 | 50 |
| 11 | 63 | 42 |
| 12 | 58 | 28 |
| 13 | 53 | 24 |
| 14 | 53 | 18 |
| 15 | 44 | 19 |
| 16 | 47 | 14 |

**Table 2**. Percentage of mothers who accessed each element of the intervention at least once each week (n=72).

| Intervention week | Percentage | | | |
| --- | --- | --- | --- | --- |
|  | Chat | Me & Baby | Timeline | Resources |
|  |  |  |  |  |
| 1 | 75 | 67 | 64 | 60 |
| 2 | 76 | 49 | 54 | 43 |
| 3 | 65 | 42 | 43 | 40 |
| 4 | 72 | 28 | 28 | 21 |
| 5 | 65 | 35 | 36 | 29 |
| 6 | 67 | 14 | 22 | 25 |
| 7 | 67 | 19 | 31 | 19 |
| 8 | 61 | 25 | 29 | 21 |
| 9 | 67 | 26 | 32 | 25 |
| 10 | 67 | 25 | 26 | 25 |
| 11 | 63 | 8 | 22 | 19 |
| 12 | 58 | 14 | 19 | 4 |
| 13 | 53 | 10 | 19 | 17 |
| 14 | 51 | 13 | 14 | 13 |
| 15 | 44 | 10 | 11 | 11 |
| 16 | 47 | 11 | 19 | 10 |

**Table 3**. Percentage of mothers who reported the helpfulness of the intervention at the 8 month assessment (n=59^a^).

| Item | Percentage | | |
| --- | --- | --- | --- |
|  | Helpful^b^ | Unhelpful^c^ | No opinion |
|  |  |  |  |
| Learning about your baby’s development | 81 | 7 | 12 |
| Getting help and support from CaFHS nurse | 80 | 5 | 15 |
| Getting help and support from mothers | 78 | 7 | 15 |
| Learning about your baby’s mood/behaviour | 73 | 5 | 22 |
| Learning to care for your baby | 71 | 5 | 24 |
| Learning to care for your emotional health | 64 | 10 | 25 |
| Confidence in caring for your baby | 63 | 10 | 27 |
| Confidence in caring for your emotional health | 63 | 10 | 27 |
| Finding accurate information on the internet | 54 | 8 | 37 |
| Learning about available community services | 53 | 8 | 39 |
| Monitoring your own/your baby’s mood | 42 | 17 | 41 |

^a^ Missing data for one participant who did not use any of the app features. Total number of participants who complete the 8 month assessment is n=60.

^b^ Responses of ‘helpful’ and ‘very helpful’ are combined in this column; ^c^ Responses of ‘unhelpful’ and ‘very unhelpful’ are combined in this column.

**Table 4**. Percentage of mothers who reported the eMums app as being ‘user-friendly’ at the 8 month assessment (n=60).

| Item | Percentage | | |
| --- | --- | --- | --- |
|  | Easy^a^ | Difficult^b^ | Not used |
|  |  |  |  |
| Reading parenting information | 85 | 8 | 7 |
| Reading emotional health information | 85 | 7 | 8 |
| Talking with mothers/CaFHS nurse in Chat | 71 | 20 | 8 |
| Private messaging CaFHS nurse | 58 | 13 | 28 |
| Finding contact details of support services^*^ | 58 | 14 | 29 |
| Finding useful apps/websites suggestions | 57 | 17 | 27 |
| Recording your baby’s mood | 50 | 18 | 32 |
| Recording your mood/thoughts/activities | 48 | 22 | 30 |
| Recording developmental milestones | 48 | 18 | 33 |
| Viewing moods/thoughts/activities graph | 27 | 27 | 47 |
| Creating a video of milestones | 20 | 20 | 60 |

*Note:* n=60 is based on the number of participants who completed the 8 month assessment.

^a^ Responses of ‘easy’ and ‘very easy’ are combined in this column; ^b^ Responses of ‘difficult’ and ‘very difficult’ are combined in this column; ^c^ Missing data for one participant, n=59
