# Supplementary material for: The Effectiveness of an App-Based Nurse-Moderated Program for New Mothers With Depression and Parenting Problems (eMums Plus): Pragmatic Randomized Controlled Trial
Source: J Med Internet Res. 2019 Jun 4;21(6):e13689. doi: 10.2196/13689 (PMC6682297; doi:10.2196/13689)
Supplement: Multimedia Appendix 4 [file jmir_v21i6e13689_app4.docx]

**Intervention app components**

1. *Chat:* It contains a chat room where mothers post questions and nurses can reply with posts and comments visible to all group members in a similar format to Facebook. Mothers can also reply and answer each other’s questions. The parenting and emotional health curriculum is posted on the chat room 2 times a week for mothers. Furthermore, the nurse posts additional content depending on the needs of her group.
2. *Resources:* It contains short articles and activities on parenting and emotional health that make up the eMums plus curriculum, as well as additional information about other topics that may be useful for mothers. This is available for mothers to search as required if they are looking for accurate CaFHS-endorsed information on a particular topic. Mothers are able to post topics from the resources section into the chat page if they want to share information with the group.
3. *Timeline:* It provides a list of child development milestones and health reminders that provides guidance to mothers appropriate to their baby’s age during the intervention. Mothers can record these items as ‘completed’ on the eMums plus app. Nurses can also view the timeline to assess whether children have completed health checks and are meeting developmental milestones.
4. *Me & Baby*: Mothers can also access a maternal and infant “mood-rater” that allows mothers to monitor their own mood and nurses to track mothers’ and infants’ moods over time. It also contains an events calendar displaying topics that nurses discuss and other material relevant to the functioning of the group.
